# Extracellular Vesicles in Heart Disease: Excitement for the Future ?

**DOI:** 10.5772/58390

**Published:** 2014

**Authors:** Kirsty M. Danielson, Saumya Das

**Affiliations:** 1 Cardiovascular Institute, Beth Israel Deaconess Medical Center, Boston, MA

**Keywords:** Exosome, Microvesicle, Apoptotic Bodies, Cardiovascular Disease

## Abstract

Extracellular vesicles (EV), including exosomes, microvesicles and apoptotic bodies, are released from numerous cell types and are involved in intercellular communication, physiological functions and the pathology of disease. They have been shown to carry and transfer a wide range of cargo including proteins, lipids and nucleic acids. The role of EVs in cardiac physiology and heart disease is an emerging field that has produced intriguing findings in recent years. This review will outline what is currently known about EVs in the cardiovascular system, including cellular origins, functional roles and utility as biomarkers and potential therapeutics.

## 1. Introduction

Cardiovascular disease (CVD) is a significant contributor to morbidity, mortality and health-care expenditures in the United States. With an improvement in earlier detection of disease, increased focus on primary prevention and better treatment of cardiovascular diseases, there has been a steady decline in mortality attributable to CVD over the past two decades. Nonetheless, the burden of CVD remains extremely high, with 32.3% of all U.S deaths directly attributable to CVD [1]. Additionally, with the decrease in acute mortality after diseases like acute coronary syndrome, treatment of cardiovascular conditions including heart failure (the late sequelae of myocardial infarctions) now accounts for an ever-increasing fraction of health-care expenditure, with an estimated 183,000,000,000 dollars spent in 2009 [1]. Unfortunately, the development of novel therapies to treat CVD has stagnated in the past decade. In light of this, there is an urgent need for an improved mechanistic understanding of heart disease pathogenesis, the identification of novel targets and innovative strategies to deliver therapies.

While cardiomyocytes (CMs) comprise the majority of the volume of the heart, multiple other cell types, including fibroblasts (which comprise 90% of the non-myocyte cells that make up 5-10% of cardiac tissue), endothelial cells and vascular smooth muscle cells make up the remainder of the heart and play an important role in cardiac function, both in health and in disease [2]. Recently, investigators have turned their attention to elucidating mechanisms of communication between these diverse cell-types, particularly as it relates to disease pathogenesis. Extracellular lipid-bilayer enclosed particles have garnered recent attention as mediators of signalling between cell types [3, 4], particularly in the field of immunology and oncology, where their role in various processes including intercellular communication [5], immune induction [6], neoangiogenesis [7] and antigen presentation [8] have been described.

Investigations into the function of these particles, which include exosomes as well as microvesicles (collectively referred to as extracellular vesicles or EVs, see definitions below), are in their infancy in cardiovascular biology. However, several recent studies suggest that these particles may not only play an important role in intercellular communication in the cardiovascular system, but may also have clinical utility as biomarkers in CVD and could potentially be utilized as vehicles for targeting therapies. This review will focus on the current state of knowledge about the role of EVs in the cardiovascular system, including characteristics of cardiac EVs and their mechanistic function, in addition to their potential use in the clinical arena.

## 2. Characterization of Cardiac Extracellular Vesicles

**Figure 1:**
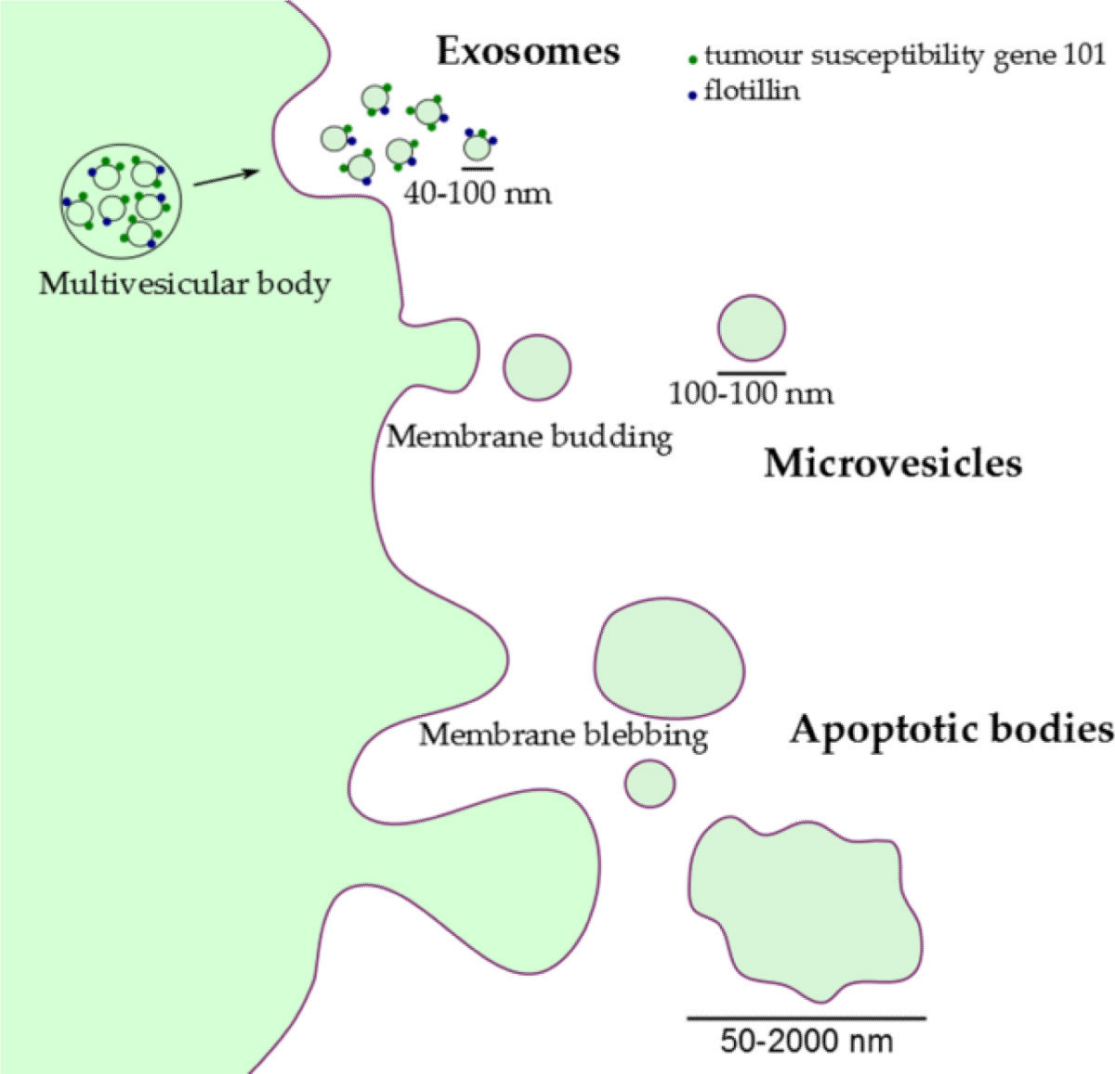
Extracellular vesicle secretion

Extracellular vesicles (EVs) consist of several distinct entities, namely exosomes, microvesicles and apoptotic bodies, which are differentiated by their mechanisms of biogenesis and secretion (figure 1). Exosomes are derived from the endolysosomal pathway, stored in multivesicular bodies and are released from cells through fusion of these multivesicular bodies to the cell membrane [9]. They are approximately 40-100 nm in diameter and investigators have proposed several specific surface markers, including tumour susceptibility gene 101 and flotillin, which identify exosomes [9]. Microvesicles, in contrast, are generally larger in size, ranging from 100-1000 nm in diameter, are formed through budding of the cell membrane and thus are heavily enriched in phosphatidylserine with a membrane composition that resembles that of the parent cell membrane [10]. Exosomes and microvesicles contain many cellular components capable of effecting intercellular communication on neighbouring and distant cells. These include cytoplasmic and membrane proteins [11], mRNA and microRNA (miRNA) [3], other non-coding RNA [12] and lipids [13]. Finally, apoptotic bodies (50-2000 nm) result from blebbing of the surface of the apoptotic cells and contain cell organelles and nuclear fractions, including non-coding RNAs that are specific to the nucleus or nucleolus [14].

Investigators have described detailed characterizations of the contents of EVs released by tumour cells [15], cells of the immune [16] and neural systems [17] and reviewed elsewhere. Much of the recent work has focused on the non-coding, particularly the miRNA, content of EVs. Of particular interest is work that notes that the miRNA profile of EVs is not representative of the parent cell miRNA profile, raising the possibility of selective export of certain miRNAs into EVs [3]. This observation was supported by a subsequent study demonstrating that certain miRNAs present in EVs were not detectable in the parent tumour cell, thereby suggesting that these miRNAs may have been transcribed specifically for transport in EVs [18]. At the current time it is not known if a similar process of selective transport of miRNAs into EVs occurs in the cells that comprise the cardiovascular system.

A significant obstacle in this rapidly developing field is the lack of standardized protocols for isolating each of these components of EVs. Detailed characterization of EVs using proteomics [19], lipidomics [13] and RNA-seq [20] has also been hindered by the diversity of the cellular origins of EVs in biospecimens, coupled with the complexity of their contents. Hence published studies have shown considerable divergence in their findings, with a lack of agreement about standardized surface or content markers for each of these components of EVs. Current isolation methods for EVs include immunoaffinity capture, differential centrifugation, the use of gradients and specialized kits produced by vendors. These isolation methods rely on different characteristics of EVs and therefore it is possible that different subpopulations of EVs will be isolated, depending on the exact technique deployed. Additionally, significant differences in EV viability have been found between different preparations [21]. EVs produced using ExoQuick (System Biosciences) were extremely robust and withstood changes in temperature and pH for up to 18 h. By contrast, EVs produced using previously published methods for clinical utility (ultrafiltration followed by ultracentrifugation through a sucrose/deuterium oxide column [22]) began degrading and releasing their contents within 2 h [21]. Therefore, differences in methods used for EV isolation need to be addressed when comparing the findings from different studies.

Differences in EVs from different cell types and species should also be taken into consideration. It is likely that EVs of different origins express different surface markers; therefore, selection of EVs based on specific markers, obtained through methods such as immunoaffinity, could result in the study of EV subpopulations that exclude some biologically relevant carriers. Future detailed characterization of EV subpopulations could result in the ability to specifically isolate subpopulations of interest, which could prove to be a valuable research tool.

The study of EVs in the cardiovascular field is beset by these same complexities. Most studies have relied on ultracentrifugation/ultrafiltration techniques to isolate EVs; however, some studies call the isolated entities *exosomes*, while others refer to them as *extracellular membrane vesicles* (EMVs). Most of these studies do not investigate the biogenesis of the isolated EVs or characterize their size and composition in detail; hence, in our opinion, it would be better to classify to them as EVs. In this review, we will therefore refer to these membrane-bound extracellular vesicles as EVs, regardless of how they were classified in the initial publication.

## 3. Origin of EVs in the Cardiovascular System

CMs and endothelial cells have both been shown to secrete EVs [23, 24]. Like EVs from other cell types, they contain a variety of proteins and RNAs from the parent cell. Proteomic analysis of EVs (which were classified as exosomes in this study) from adult rat CMs revealed some common proteins with previous studies from different cell origins, including glyceraldehyde-3-phosphate dehydrogenase (GAPDH) and actin [21]. However, many other proteins previously found in EVs in other studies, including HSP90 and the tetraspanins, were not seen in the CM-derived EVs [21]. In contrast, another study did identify previously reported tetraspanins in EVs derived from human microvascular endothelial cells in culture [24]. It should be noted that many of the earlier proteomics studies focused on EVs secreted from human tumour cells. The differences in protein expression between the EVs of cardiovascular origin may reflect differences in both cell type- and species- specificity of EV characteristics.

Currently, no studies have directly demonstrated the release of EVs from cardiac fibroblasts. However, in neonatal rat cell culture, paracrine factors from cardiac fibroblast-cultured media produced changes in CM electrophysiology [25] and improved CM viability in hypoxic conditions [26]. Investigating whether EVs are implicated in mediating these paracrine factors would be an interesting area for future research.

Another interesting finding from proteomics studies is that stimulation of cell cultures with different stressors produces EVs with differing contents. This was observed in both adult rat CMs in response to hypoxia/reoxygenation or ethanol treatment [21] and in human microvascular endothelial cells in response to hypoxia and TNF-α treatment [24]. In endothelial cell-derived EVs, several of these findings, which were confirmed with immunblot analysis, included an increase in lysyl oxidase like-2 in EVs from hypoxia-treated cells and an increase in ICAM-1 in EVs derived from TNF-α treated cells. In addition, differences in mRNA expression in endothelial cells in response to these stressors were observed [24]. These studies raised the possibility that EVs may play a role in inter-cellular transfer of stress signals and therefore play an important role in disease pathogenesis.

## 4. Functional Role of EVs in Cardiovascular Biology and Disease

**Figure 2:**
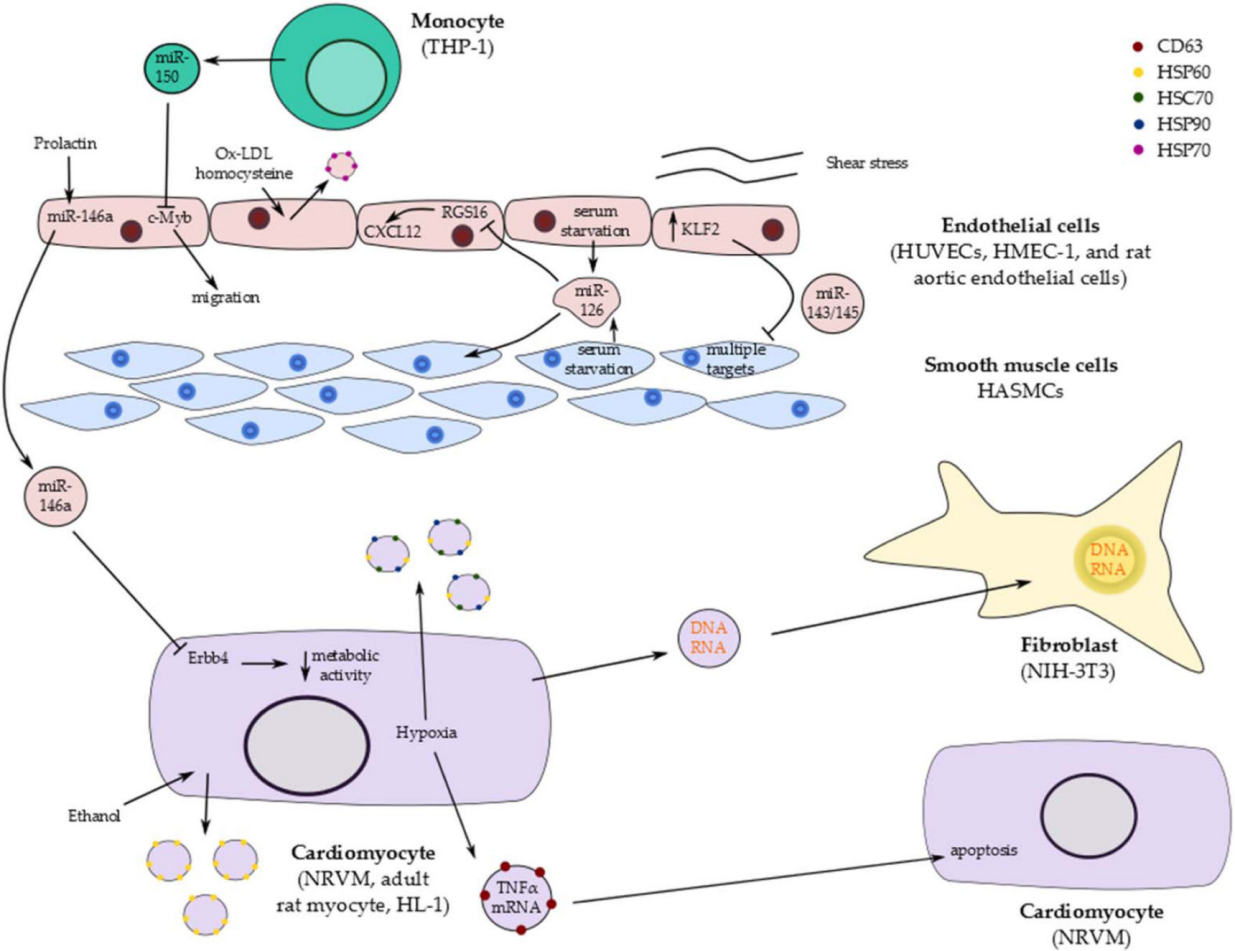
Summary of reported EV release from cardiac cells in culture and their functional effects. EV release has been reported in numerous cell lines and primary cultures, both at baseline and in response to stimuli. Transfer of EVs between cell types has been demonstrated as well as delivery of cargo. HUVEC: human aortic endothelial cell; HMEC-1: human microvascular endothelial cell; HASMC: human aortic smooth muscle cell; NRVM: neonatal rat ventricular myocyte.

A summary of reported EV release from different cell types in vitro and their functional effects is represented in figure 2.

### 4.1 Cardiomyocyte-derived EVs

EV release from CMs (classified as exosomes in this study) was first reported by Gupta and Knowlton in primary cultures of adult rat CMs, in which EVs were isolated using classical differential centrifugation and ultracentrifugation techniques [27]. In this study, EVs were released from CMs at baseline, but their release was tripled (as measured by acetylcholine esterase activity [28]) in response to brief hypoxia. HSP60, which had not been previously found in EVs from other cell types, was determined to be a component of these EVs, in addition to the previously described markers HSC70 and HSP90 [27]. The release of EVs containing TNF-α in response to hypoxia has also been reported [29]. In neonatal rat CMs, hypoxia induced increases in TNF-α mRNA within the cell and caused the release of the 26 kDa transmembrane form of TNF-α in CD63^+^ EVs. Exposure of healthy CMs to these EVs induced apoptosis. The authors suggested that although TNF-α is not normally produced in the heart, CMs can produce it under stress, package it into EVs and thereby affect apoptosis in neighbouring cells [29]. Transfer of TNF-α via EVs may therefore provide a mechanism by which stressed cells participate in the propagation of an inflammatory response.

A subsequent study also showed the release of HSP60-containing EVs from CMs in response to ethanol exposure [21]. The authors argued that this mechanism may be reactive oxygen species-mediated, as supported by the finding of increased Cell-Rox red staining in the ethanol treated cells compared to controls. The implications of the HSP60 in the EVs derived from these cells remains unclear. HSP60 is found in both the mitochondria and cytosol of CMs and has a complex role in apoptosis and cell signalling. Increases in HSP60 have been reported to prevent apoptosis [30]; however, translocation of HSP60 to the plasma membrane has also been reported to increase apoptosis [31] and extracellular HSP60 (which has been detected in rodent and human plasma) appears to contribute to myocardial injury during ischemia [32]. In the current study, HSP60 was mono-ubiquitinated - associated with lipid raft structures - and tightly bound to the membrane surface with the EVs. The authors suggested that EV-bound HSP60 functions as an intercellular signal without causing toxicity due to its encapsulation within the membrane, although these assumptions were not directly proven [21]. Uptake of these exosomes by other cell types and the subsequent functional effects were not investigated and could be the subject of future studies.

In contrast to the studies already described, where the fate of the secreted exosomes was not directly investigated, a recent study demonstrated the transfer of DNA and RNA from CMs to fibroblasts [23]. EVs released by HL-1 cells (an immortalized atrial myxoma cell line) were found to contain DNA and RNA, as labelled with acridine orange. When added to NIH-3T3 cultures, the acridine orange-labelled EVs were shown to transfer the acridine orange staining into NIH-3T3 fibroblasts, often into their nuclei, thereby demonstrating EV-mediated transfer of genetic information from CMs to fibroblasts. Cargo present within the EVs included ribosomal RNA and mRNA coding for proteins and transfer of this produced changes in gene expression within the fibroblasts [23]. However, the exact mRNAs or miRNAs that affected the changes in gene transcription in the recipient cells were not identified. Furthermore, since NIH-3T3 cells are not a cardiac- specific fibroblast cell line, the intriguing idea that there may be differences between uptake of CM EVs in cardiac fibroblasts and in fibroblasts from other organs was not explored.

### 4.2 Stem cell-derived EVs

In contrast to the limited data on EV secretion by mature CMs, considerably more data exist for the release of EVs by cardiac progenitor cells (CPCs) and other types of stem cells. Spurred by the observation that beneficial effects of transplanted CPCs and other types of stem cells are often out of proportion to the number of surviving engrafted donor cells, investigators have proposed the ‘paracrine hypothesis’. This hypothesis postulates that in addition to possible direct differentiation of the engrafted donor stem cells to the appropriate cell lineages, paracrine factors released by the donor stem cells may contribute to improvements in heart function [33, 34]. This has been confirmed by numerous studies that have shown that conditioned media from stem cells can enhance CM survival after hypoxic injury [35], induce angiogenesis in infarcted myocardium [36] and reduce infarct size in both mouse [37, 38] and porcine [36, 39] models of myocardial ischemia reperfusion injury.

In many cases, these paracrine factors are packaged within EVs. Conditioned media from mesenchymal stem cells (MSC) has been shown to reduce infarct size and improve cardiac remodelling in a pig model of myocardial ischemia/reperfusion and some of this activity was contained within EVs isolated by traditional ultracentrifugation methods [36]. Similarly, MSC-derived EVs inhibited vascular remodelling and attenuated hypoxia-induced pulmonary hypertension, probably by inhibiting STAT-3 signalling in endothelial cells [40]. At least some of the paracrine effects of stem cells in recruiting endothelial cells and mediating angiogenesis appear to be contained in flotillin-positive 100 nm extracellular vesicles (isolated by differential ultracentrifugation) [33]. While an extensive characterization of the contents of EVs released from the different types of stem cell described in these studies has not been conducted, much of the recent attention has focused on the role of miRNAs packaged in these entities [41]. Following in the footsteps of cancer biology, future studies will no doubt pinpoint exact miRNAs that are packaged and released in EVs and may affect surrounding and distant cells.

### 4.3 Endothelial cell-derived EVs

Endothelial cells have been found to be a potent source of EVs and several recent studies have shed light on the role of EVs in mediating communication between vascular endothelial cells, the underlying smooth muscle cells and the circulating immune cells in the pathogenesis of atherosclerosis.

At high levels, Ox-LDL and homocysteine are risk factors for atherosclerosis and coronary artery disease [42, 43]. In contrast, high levels of circulating HSP70 have been suggested to be concurrent with a lower risk of vascular disease [44]. Both Ox-LDL and homocysteine induced the release of exosomes containing HSP70 from rat aortic endothelial cells [45]. Interestingly, HSP70 activation of monocytes preceded their adhesion to endothelial cells. Heat shock proteins are generally accepted to be protective within the cell; however, extracellular HSPs may be toxic or trigger inflammatory responses. Whether monocyte activation by EV-derived HSP70 fulfils a protective or deleterious role remains unclear: it could act as an early immune response to vascular damage, or conversely play a role in early atherosclerotic lesion formation via monocyte infiltration of the endothelium.

Communication between monocytes and endothelial cells via EVs occurs in both directions. EVs released from the monocytic cell line THP-1 were capable of transferring miR-150 to human microvascular endothelial cells (HMEC-1) [46]. Similarly, miR-150 enriched EVs isolated from human plasma were transferred to HMEC-1 cells; however, it was not determined whether they originated specifically from monocytes, from another cell type, or from multiple cell types. Treating THP-1 cells with lipopolysaccharide, hydrogen peroxide, advanced glycation end products and oleic acid/palmitic acid caused the release of EVs with markedly different expression patterns and levels of miRNAs, indicating specificity in the packaging and release of miRNAs depending on the exact stimulus. The transfer of miR-150 to HMEC-1 cells via EVs induced migration of these cells, presumably through the knock-down of c-Myb [46].

In addition to communication between circulating monocytes and endothelial cells, the transfer of information between endothelial cells and smooth muscle cells may play an important role in atherosclerotic plaque formation. Disorganization and dysfunction of the smooth muscle cells underlying endothelium in the vasculature occurs during plaque formation and contributes to pathogenesis [47]. Kruppel-like factor 2 (KLF2) is a shear-responsive transcription factor believed to be atheroprotective by preventing the formation of atherosclerosis [48]. Increased expression of KLF2 in human umbilical vein endothelial cells (HUVECs) via lentiviral transduction or exposure to shear stress causes the release of miR-143/145 in EVs [49]. Interestingly, miR-143/145 was increased approximately 30-fold in EVs compared to only 10-fold in the cells, suggesting targeted transcription of the miRNAs specifically for release. Co-culture of HUVECs with human aortic smooth muscle cells (HASMCs) resulted in the transfer of miR-143/145 to the smooth muscle cells and the reduction of multiple target mRNAs. Most importantly, transfer of miR-143/145 from endothelial cells to smooth muscle cells appears to be atheroprotective in vivo. Injection of EVs from KLF2-transduced mouse endothelial cells into ApoE−/− mice produced a twofold reduction in aortic lesion area compared to controls [49].

Finally, evidence of EV-mediated transfer of information between endothelial cells and CMs was recently demonstrated [50]. While investigating the mechanistic basis of peripartum cardiomyopathy, it was found that a fragment of prolactin (16K PRL) could induce the expression of miR-146a in endothelial cells, leading to inhibition of angiogenesis. Furthermore, 16K PRL could also induce the release of miR-146a-loaded EVs from endothelial cells. These EVs were found to be absorbed into CMs where they decreased metabolic activity and down-regulated target molecules Erbb4, Notch1 and Irak1. Signal transduction by miR-146a packaged in EVs appeared to be important in disease pathogenesis, as evidenced by amelioration of the disease phenotype by antagonism of miR-146a [50].

The majority of studies on the function of informational transfer via EMVs have focused on EVs; however, there is evidence that apoptotic bodies also play an important role in intercellular communication [51]. Endothelial and smooth muscle cell apoptosis have been implicated in the pathology of atherosclerosis and apoptotic bodies are known to carry an array of DNA fragments, RNA and proteins. Indeed, significant enrichment of miR-126 in apoptotic bodies released from serum-starved HUVECs and HAMSCs has been demonstrated [14]. Application of these apoptotic bodies to healthy HUVECs induced increased expression of the chemokine CXCL12, which is known to recruit progenitor cells and counteract apoptosis in response to arterial injury. In addition, HUVEC-derived apoptotic bodies induced increased CXCL12 expression in HASMCs and mouse aortic endothelial cells. The increase in CXCL12 expression was attributed to suppression of RGS16 by miR-126. This indicates that the release of miR-126 enriched apoptotic bodies may non-specifically signal to neighbouring vascular cells to promote cell survival. Most importantly, miR-126-enriched apoptotic bodies derived from atherosclerotic plaques of human patients and administered to ApoE−/− mice reduced plaque area and macrophage content in the aortic root of mice [14].

In summary, the studies described above provide examples of the functional role of EVs in mediating transfer of information and signal transduction between endothelial cell types and other important cell types in the cardiovascular system. While considerable literature exists documenting the important role played by EVs in communication between tumour cells and endothelial cells, the last two years have seen some progress in describing this important mechanism in the cardiovascular system and may spur the development of novel biomarkers as well as therapeutic targets.

## 5. EVs as Biomarkers in the Cardiovascular System

As previously described, miRNAs and signalling proteins specific to the parent cell may be selectively packaged into EVs and released into biofluids including plasma, urine, saliva and cerebrospinal fluids. In the case of tumours, these EVs may contain markers specific for these tumours. Apart from their possible functional role in altering the tumour microenvironment, the sequestration of the RNAs and proteins within the EVs protect them from degradation by RNAses and proteinases. These characteristics make EVs attractive candidates for studying novel biomarkers. This has been extensively investigated in the fields of oncology and neurology [52, 53]. In cardiovascular biology, there has been considerable excitement in studying plasma miRNAs as novel diagnostic and prognostic biomarkers. While several of the biomarkers described so far coincidentally reside within EVs, the use and characterization of EVs as biomarkers has not been systematically investigated.

Significantly increased serum levels of miR-1 and miR-133a has been found in patients with unstable angina pectoris, Takotsubo cardiomyopathy and acute myocardial infarction [54]. Stimulation of a rat myoblast cell line (H9c2) with the calcium ionophore A23187 induced the release of EVs containing miR-133a. Interestingly, miR-133a levels were decreased in infarcted heart tissue in a mouse model of myocardial infarction and release of EVs from H9c2 cells was associated with cell death. This led the authors to conclude that circulating miR-133a is released in response to cardiac injury and could be used as an indicator of CM death [54]. It is not known, however, whether these miRNAs contribute to pathology and cell death, or whether they may play some compensatory role to prevent the death of surrounding cells.

Sera miR-192, miR-194 and miR-34a were identified as prognostic biomarkers in patients with acute myocardial infarction. They appeared to predict the development of clinical heart failure and ventricular remodelling within one year of acute myocardial infarction onset [55]. Interestingly, all these miRNAs were regulated by p53 and packaged in CD63+ EVs. They appear to have a deleterious role as transfer of these secreted miRNAs to cultured myoblasts from embryonic rat heart accelerated cell death, while cell viability was increased by knockdown of the three miRNAs [55].

Endothelial cells have been described as an important source of miRNAs packaged into EVs. MiR-146a, as described above may play an important role in the pathogenesis of peripartum cardiomyopathy by mediating signalling between endothelial cells and CMs [50]. As noted in the previous section, miR-146a is packaged in EVs and released by endothelial cells and in human subjects was specifically diagnostic for peripartum cardiomyopathy, compared with idiopathic dilated cardiomyopathy [50].

There have been numerous studies in recent years on putative miRNA biomarkers in cardiovascular disease, as summarized in [56]. While they have not all been identified as membrane-enclosed entities, the demonstration of EV release from cardiac cells justifies additional investigation into this specific subclass of miRNAs. Further evidence supporting the origin of some of these biomarker plasma miRNAs in the heart came from work that showed a concentration gradient of some of the miRNAs recognized as biomarkers of myocardial injury, suggesting secretion from myocardial cells [57]. Nonetheless, the exact cellular source of these miRNAs, the mechanisms of packaging and secretion and their functional roles remain a matter of active investigation. Thus, there is a rapidly expanding field in identifying and validating novel diagnostic and prognostic biomarkers in cardiovascular biology. A large majority of these appear to be packaged in EVs and therefore protected from degradation by circulating RNAses. However, the detailed characterization of EVs released into the circulation by myocardial cells has lagged behind and now appears poised for further fruitful investigation.

## 6. EVs as Therapeutic Modalities in Cardiovascular Diseases

In addition to their use as biomarkers, EVs could potentially be used in novel therapeutics for cardiovascular disease. As discussed in the previous section, several cellular pathways that are targeted by EVs originating from cardiac cells have now been identified. Possible therapeutic options in cardiovascular disease involve targeting the EVs containing RNA, DNA or proteins as mediators of signalling pathways involved in disease pathogenesis. Blocking the delivery of EV cargo to cells could be achieved in several ways, including the inhibition of vesicle release, uptake, or formation [58]. While no studies have yet described the use of such strategies in cardiovascular disease, they have been studied to some extent in cancer therapy. For example, amiloride-induced inhibition of ceramide, a lipid component of sphingomyelin, can reduce vesicle yields and it enhanced the efficacy of the chemotherapeutic drug cyclophosphamide in mice [59]. A problem with this strategy, however, is its potential off-target effects. It is now known that many different cell types produce EVs and these contain a number of biologically active molecules. Therefore a blanket disruption in vesicle biology could produce many unwanted effects.

Alternatively, EVs could be used as therapeutic delivery tools. Since EVs are capable of carrying a wide range of cargo and transferring exogenous nucleic acids, they are an attractive option for gene therapy and drug treatments. In addition, some EVs have innate therapeutic activity. As discussed previously, transfer of EVs from stem cells has been shown to have beneficial effects in animal models of cardiovascular disease [36, 37, 39]. This suggests two possible strategies for using EVs as a therapeutic: deliberate packaging of specific components into vesicles for targeted delivery and use of endogenously-produced stem cell EVs without designed contents. Proof of concept for the specific packaging and targeting of EVs has been provided by recent studies including [60]. In this study, dendritic cells were engineered to express the exosomal membrane protein Lamp2b fused to neural specific rabies virus glycoprotein peptide. EVs derived from these cells were shown to deliver GAPDH siRNA specifically to the brain of mice following intravenous injection [60]. Although a similar study is yet to be conducted on the cardiovascular system, this does provide encouraging evidence for the viability of EVs as a therapeutic modality. Furthermore, methods for the large scale production of clinical grade EVs from dendritic cells [22] and mesenchymal stem cells [61,62] are currently being developed and offer considerable promise. With continuing research on the role and regulation of EVs in cardiovascular disease, they could offer a novel therapeutic tool in the future.

## 7. Conclusion

In summary, EVs released from cardiovascular cells have been shown to transfer to different cell types and cause changes in RNA and protein expression that have significant downstream functional effects. Functional changes reported to date appear be both protective and deleterious and may play an important role in normal physiology or disease pathogenesis. Regardless of whether the observed effects of EV transfer produce positive or negative effects, EVs provide an attractive target for potential therapies and use as novel biomarkers.

There is considerable uncharted territory that is yet to be explored by investigators. EVs have been shown to express surface ligands such as integrins that can interact with proteins of the extracellular matrix, where they may be stored as repositories of signalling. Alterations in the extracellular matrix, as may occur with infarction or haemodynamic changes resulting from heart failure may result in the ‘uncovering’ of these EVs and their signalling capabilities. This hypothesis has been explored in cancer biology, but remains to be examined in heart disease.

Detailed characterization of EVs released by the components of the cardiovascular system both at baseline and with stress may provide additional insight into novel intercellular signalling mechanisms. As attempts to undertake these ventures gather pace, we may be on the threshold of a new era of mechanistic understanding of heart disease.
